# Supplement intake variation, weight, and body condition change in yearling heifers grazing late-summer dryland pastures with Rumax BoviBox vs. Rumax BoviBox HM protein supplements^1^

**DOI:** 10.1093/tas/txaa130

**Published:** 2020-12-22

**Authors:** Tyrell P McClain, Samuel A Wyffels, Shay R Larsen, A Luka Müller, Noah G Davis, Boone H Carter, Jan G P Bowman, Darrin L Boss, Timothy DelCurto

**Affiliations:** 1 Department of Animal and Range Science, Montana State University, Bozeman, MT; 2 Northern Agricultural Research Center, Montana State University, Havre, MT; 3 PerforMix Nutrition Systems, Nampa, ID

## INTRODUCTION

Beef production is an important facet to the global economy and is the top agricultural commodity in Montana. In 2019, there were 380,000 replacement heifers developed in Montana, representing 15% of all cattle in the state (NASS, 2019). Proper heifer development needs to be economically managed without sacrificing heifer performance (Funston and Deutscher, 2004). Heifers developed in forage-based systems may provide economic advantages compared to heifers developed on high-quality diets in confinement (Funston et al., 2007; Mulliniks et al., 2013). In addition, the high costs of feed associated with developing heifers have encouraged producers to search for alternative methods of reducing the reliance of harvested feeds (Adams et al., 1986; Funston et al., 2007). Effective use of forage-based heifer development systems requires strategic supplementation to offset nutrient deficiencies (Bowman and Sowell, 1997; DelCurto et al., 2000; Bodine et al., 2001).

Producers who are dependent on forage resources for feed must develop strategies that maximize forage use while minimizing supplemental inputs in order to reduce costs and maintain acceptable levels of beef cattle production (DelCurto et al., 2000). Protein blocks and/or salt-limited supplements are a convenient product for producers because they can increase low-quality forage intake and improve animal performance (Horn and McCollum, 1987; McCollum and Horn, 1990). Past research has suggested that these supplementation strategies may be limited because of intake variation (Bowman and Sowell, 1997). Livestock managers and supplement manufactures often manipulate salt content, texture, and bitterness in an effort to regulate supplement intake. An approach presumed to influence protein block intake is to increase Mg levels of the supplement to subsequently increase bitterness and hardness. However, research evaluating self-fed, free-choice supplements with differing Mg levels in forage-based systems are limited.

Advances in the technology of self-contained feed systems have made it possible to acquire accurate, individual feed intake data that includes intake behavior attributes, such as time spent feeding, number of feeding visits per day, and total intake per visit (Reuter et al., 2017; Williams et al., 2018a, 2018b). Therefore, the objective of this study was to evaluate the effect of Mg levels of two forms of a protein block supplement BoviBox and BoviBox high magnesium (HM) on intake behavior and performance of yearling replacement heifers grazing late-summer dryland pasture. Therefore, we hypothesize that supplement form (Mg level) will influence supplement intake behavior and animal performance.

## MATERIALS AND METHODS

The care and use of cattle in this study were approved by the Institutional Animal Care and Use Committee of Montana State University (ACUP #2018-AA09). All animals used in this study were provided by the Montana Agricultural Experiment Station.

This study was conducted at Montana State University’s Fort Ellis Research Farm located 8 km east of Bozeman, MT (45˚38’N, 110°58’W, 1,500 m elevation). Annual precipitation is 470 mm, with 55% occurring during the growing season (May through September). Vegetation is dominated by smooth brome (*Bromus inermis*) and Kentucky bluegrass (*Poa pratensis*).

Fifty-nine yearling commercial Angus heifers (428 kg) grazed five dryland pastures (average 10.5 ha) over 84 d between July 22 and October 14, 2019. All cows were weighed and body condition scored (BCS; NRC, 2016) following a 16-h shrink prior to the initiation of the study and again at days 42 and 84. Heifers were stratified by BCS and body weight (BW) and, then, within stratum, randomly assigned to one of the following supplement treatments: 1) free-choice Rumax BoviBox protein block [Table 1; 30% crude protein (CP), 23% salt; *n* = 29] and 2) free-choice Rumax BoviBox HM protein block (Table 1; 28.7% CP, 23% salt; *n* = 30). Target intake for both supplements was 0.45–0.91 kg/heifer/d. The BoviBox HM is designed for producers with cattle that consume the BoviBox supplement at levels that exceed the target intake.

**Table 1.** Guaranteed analysis of protein block supplements (contains not more than 9.9% and 9.7% protein from nonprotein nitrogen)

Ingredient	Rumax BoviBox	Rumax BoviBox HM
CP	30% min	28.7% min
Crude fat	1.5% min	1.45% min
Crude fiber	5.0% max	5.0% max
Calcium	1.3% min	1.3% min
	1.8% max	1.8% max
Phosphorus	0.7% min	0.7% min
Salt	23% min	23% min
		26% max
Potassium	1.5% min	1.5% min
Magnesium	1.0% min	2.5% min
Manganese	880 ppm	856 ppm min
Zinc	1,100 ppm	1,074 ppm min
Copper	220 ppm	213 ppm min
Copper (from chelate)	110 ppm	108 ppm min
Cobalt	16 ppm	15 ppm min
Iodine	25 ppm	26 ppm min
Selenium	3.3 ppm min	3.3 ppm min
	3.6 ppm max	3.6 ppm max
Selenium yeast	1.7 ppm	—
Vitamin A	40,800 IU/lb	12,000 IU/lb
Vitamin D	4,500 IU/lb	4,000 IU/lb
Vitamin E	50 IU/lb	25 IU/lb

An electronic identification (EID) tag (Allflex USA, Inc., Dallas-Ft. Worth, TX) was placed in the left ear of each heifer. Supplement was delivered in a centrally located SmartFeed Pro self-feeder system (C-Lock Inc., Rapid City, SD). The SmartFeed system contains four feed bunks mounted on scales, individually equipped with EID tag readers and locking gates. Each treatment was placed in two feed bunks and the SmartFeed system was programmed to only allow heifers access to their respective treatment. The SmartFeed system recorded individual supplement intake and time spent at feeder for each appearance at the feed bunks. Using data from the SmartFeed system, we calculated mean daily intake, mean daily intake per unit BW, mean intake rate, mean daily time spent at the feeder, and mean intake CV for each heifer for both periods of the study.

Every 14 d, 10 random 0.11-m^2^ plots were clipped in the pasture the heifers were using. All samples were weighed, composited by date, sent to a commercial laboratory, (Dairy One, Ithaca, NY) and analyzed for CP, total digestible nutrients (TDN), neutral detergent fiber (NDF), and acid detergent fiber (ADF).

Intake, intake per unit BW, intake rate, time spent at the feeder, intake CV, BW change, and BCS change were analyzed in R using generalized linear mixed models that included supplement formulation, period, and a supplement formulation × period interaction as fixed effects and individual heifer as a random intercept (R Core Team, 2019). Each heifer was considered an experimental unit. Least square means were separated using the Tukey method. Statistical significance was accepted at *P* < 0.05.

## RESULTS

The influence of supplement formulation on supplement intake behavior variables are listed in Table 3. Supplement intake (kilograms per day) displayed a treatment × period interaction (*P* < 0.01). However, within period, treatment differences were not observed (*P* > 0.05), although period intakes differed averaging 0.15 and 0.34 kg/d for days 0–42 and 42–84, respectively (*P* < 0.01). Supplement intake expressed as grams per kilogram BW per day displayed a period effect (*P* < 0.01) where supplement intake was higher in period 2 than period 1 (0.72 ± 0.04 and 0.34 ± 0.04 g/kg BW/d, respectively). Supplement intake rate (grams per minute) displayed a treatment effect where intake rate of Rumax BoviBox HM was higher than Rumax BoviBox (*P* = 0.02). In addition, supplement intake rate was 30% greater in period 2 than in period 1 (*P* < 0.01). Time spent at the feeder displayed a period effect (*P* < 0.01) where cows spent nearly twice as much time at the feeders in period 2 than in period 1. Likewise, intake coefficient of variation (CV) displayed a period effect (*P* < 0.01) where intake in period 1 was more variable than in period (218 ± 9.63 % and 163 ± 8.00 %, respectively).

**Table 2.** Forage quantity (kg/ha) and quality (%) of late-summer dryland pastures grazed by yearling heifers in Bozeman, MT^*a*^

	Production	Dry matter	TDN	CP	NDF	ADF
Pasture 1						
Day 0	3,937.3	93.9	58	8.9	57.7	37.9
Day 14	2,535.2	94.7	56	7.5	62.6	38.7
Day 28	2,094.5	97.2	56	5.3	62.4	38.1
Pasture 2						
Day 28	2,460.3	95.1	60	10.9	49.9	35.5
Day 42	1,480.8	95.3	56	6.6	61.8	39.8
Pasture 3						
Day 42	4,412.7	95.3	56	5.6	62.0	39.5
Day 56	2,611.1	96.9	57	5.5	60.5	36.3
Day 70	2,794.5	96.1	55	4.6	66.2	42.0
Pasture 4						
Day 70	2,940.5	94.2	57	10.4	60.1	41.4
Pasture 5						
Day 84	3,431.2	96.6	55	5.4	68.4	43.3

^*a*^Forage production and quality was estimated every 14 d using 0.11 m^2^ plot frames and 10 plots per sampling time.

**Table 3.** Influence of magnesium level in supplement, Rumax BoviBox vs. Rumax BoviBox HM, on supplement intake behavior of yearling heifers grazing dryland pastures

	Treatment^*a*^			*P* values		
	BoviBox	BoviBox HM	SEM^*b*^	Trt^*c*^	Pd^*d*^	Trt × Pd^*e*^
Intake, kg/cow/d				0.43	<0.01	<0.01
Period 1	0.13	0.16	0.02			
Period 2	0.36	0.32	0.02			
Intake, g/kg BW/d				0.34	<0.01	<0.01
Period 1	0.30	0.37	0.05			
Period 2	0.77	0.68	0.05			
Intake rate, g/min				0.02	<0.01	0.21
Period 1	25.0	37.9	3.87			
Period 2	41.4	46.4	4.18			
Time spent at feeder, min/d				0.63	<0.01	0.06
Period 1	3.81	4.19	0.57			
Period 2	8.07	7.27	0.59			
CV supplement intake, %				0.06	<0.01	0.26
Period 1	237.00	200.00	13.65			
Period 2	164.00	161.00	11.30			

^*a*^Treatments are 1) Rumax BoviBox and 2) Rumax BoviBox HM. ^*b*^ *n* = 30. ^*c*^Treatment main effect. ^*d*^Period main effect.

^*e*^Treatment × period interaction.

The influence of supplement formulation on BW and BCS are listed in Table 4. Change in BW was not influenced by supplement formulation (*P* = 0.89) but was influenced by period (*P* < 0.01) where the gain in period 1 was greater than period 2 (33.89 ±1.23 kg and 8.34 ± 1.33 kg, respectively). Change in BCS was not influenced by supplement formulation or period (*P* ≥ 0.89).

**Table 4.** Influence of magnesium level in supplement, Rumax BoviBox vs. Rumax BoviBox HM, on BW and body condition of yearling heifers grazing dryland pastures

	Treatment^*a*^			*P* values		
	BoviBox	BoviBox HM	SEM^*b*^	Trt^*c*^	Pd^*d*^	Trt × Pd^*e*^
Initial BW, kg	429.00	429.00	4.24	0.97	—	—
Initial BCS	5.38	5.43	0.06	0.49	—	—
Δ BW, kg				0.89	<0.01	0.21
Period 1	34.06	33.73	1.74			
Period 2	6.21	10.48	1.88			
Δ BCS	0.16	0.16	0.05	0.89	0.99	0.79

^*a*^Treatments are 1) Rumax BoviBox and 2) Rumax BoviBox HM. ^*b*^ *n* = 30. ^*c*^Treatment main effect. ^*d*^Period main effect.

^*e*^Treatment × period interaction.

## DISCUSSION

**Figure 1.**
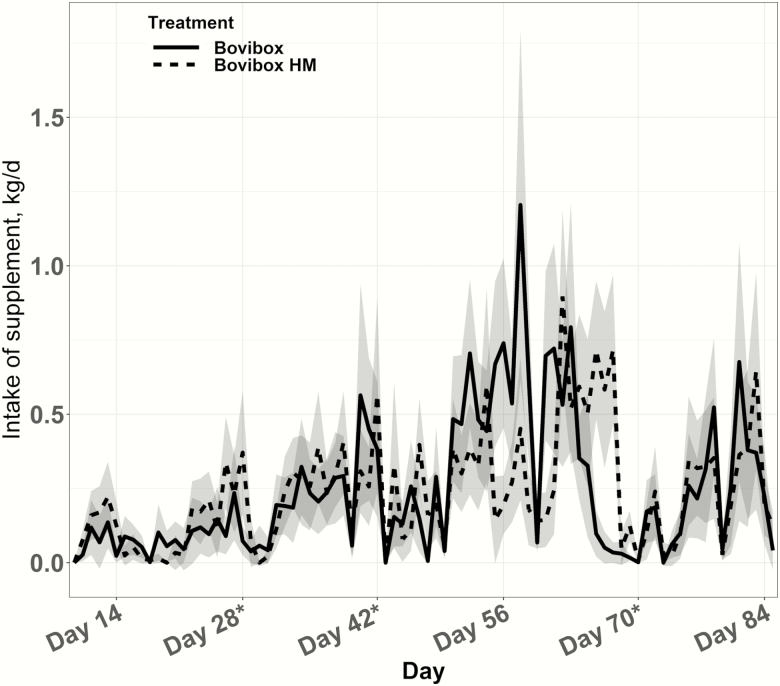
Supplement intake behavior of yearling heifers consuming BoviBox or BoviBox HM supplements over an 84-d period on dryland pastures. Asterisk (*) indicates pasture move dates over the 84-d period.

When plotting average daily intake over the 84-d trial (Fig. 1), supplement intake appeared to be influenced by pasture move dates, which, in turn, seemed to be related to forage quality/quantity for each pasture (Table 2). The quality of available forage was only marginally deficient and both forage quantity and quality differed among pastures and over the 84-d study period. Similar to our study, other researchers have observed forage quality/quantity impacts on supplement intakes with intake increasing with declining forage quality and availability (Wagnon, 1965; Ducker et al., 1981; Bowman and Sowell, 1997).

Research relating to individual animal supplement intake in extensive environments, such as pastures and rangelands, have only recently been reported. Actual intakes of salt-limited canola base supplements have been found to vary across animal age and forage quality/quantity attributes (Wyffels et al. 2018). White et al. (2019) reported supplement intake behavior with heifers on the same paddocks as our study in previous years and also found that intake was higher from days 42 to 84 compared to days 0 to 42 (1.14 vs. 0.5 kg/d). In addition, in a 2-yr winter grazing study in the mixed-grass prairie, Wyffels et al. (2020) reported higher intakes with the BoviBox supplement (year 1: 0.45–0.91 kg/d) compared to BoviBox HM supplement (year 2: <0.45 kg/d) with yearling heifers having the lowest intakes and the greatest intake variation (CV) averaging 0.24 kg/d and 200% CV, respectively. This suggests that yearling heifers in our study were not as effective at consuming BoviBox supplements and intake may be lower because of adequate forage quantity and quality.

## IMPLICATIONS

Minor effects were observed in supplement intake behavior when comparing BoviBox vs. BoviBox HM supplement treatments. However, supplement intake behavior was strongly influenced by period, which could be related to advanced stages of plant phenology where greater daily intakes and reduced intake variation were observed with declining forage quality and availability. Our study suggests that intake behavior of the free-choice (salt, bitterness, and texture) supplements changed in relation to forage quantity and quality.

## References

[CIT0001] Adams D., Nelsen T., Reynolds W., and Knapp B.. 1986 Winter grazing activity and forage intake of range cows in the Northern Great Plains. J. Anim. Sci. 62(5): 1240–1246. doi:10.2527/jas1986.6251240x.

[CIT0002] Bodine T. N., Purvis H. T. II, and Lalman D. L.. 2001 Effects of supplement type on animal performance, forage intake, digestion, and ruminal measurements of growing beef cattle. J. Anim. Sci. 79:1041–1051. doi:10.2527/2001.7941041x. 11325178

[CIT0003] Bowman J. G., and Sowell B. F.. 1997 Delivery method and supplement consumption by grazing ruminants: a review. J. Anim. Sci. 75:543–550. doi:10.2527/1997.752543x. 9051478

[CIT0004] DelCurto T., Hess B., Huston J., and Olson K.. 2000 Optimum supplementation strategies for beef cattle consuming low-quality roughages. J. Anim. Sci. 77:1–16. doi:10.2527/jas2000.77E-Suppl1v.

[CIT0005] Ducker M., Kendall P., Hemingway R., and McClelland T.. 1981 An evaluation of feedblocks as a means of providing supplementary nutrients to ewes grazing upland/hill pastures. Anim. Sci. 33(1):51–57. doi:10.1017/s0003356100025198.

[CIT0006] Funston R. N., and Deutscher G. H.. 2004 Comparison of target breeding weight and breeding date for replacement beef heifers and effects on subsequent reproduction and calf performance. J. Anim. Sci. 82:3094–3099. doi:10.2527/2004.82103094x. 15484963

[CIT0007] Funston R. N., Martin J., and Roberts A.. 2007 Heifer development–then and now. In: The Range Beef Cow Symposium XX December 11 to 13, 2007; Fort Collins, Colorado.

[CIT0008] Horn G., and McCollum F.. 1987 Energy supplementation of grazing ruminants. In: Grazing Livestock Nutrition Conference. Jackson: University of Wyoming; p. 125–136.

[CIT0009] McCollum F. III, and, Horn G. 1990 Protein supplementation of grazing livestock: a review. Prof. Anim. Sci. 6(2):1–16. doi:10.15232/s1080-7446(15)32251-8.

[CIT0010] Mulliniks J. T., Hawkins D. E., Kane K. K., Cox S. H., Torell L. A., Scholljegerdes E. J., and Petersen M. K.. 2013 Metabolizable protein supply while grazing dormant winter forage during heifer development alters pregnancy and subsequent in-herd retention rate. J. Anim. Sci. 91:1409–1416. doi:10.2527/jas.2012-5394. 23296826

[CIT0011] NASS. 2019 Montana Agricultural Statistics. Mountain region—Montana Field Office. No. LVI. 63 p. USDA’s NASS, Helena, MT.

[CIT0012] NRC. 2016 Nutrient requirements of beef cattle. Washington, DC: National Academies Press.

[CIT0020] R Core Team. 2019 R: A language and environment for statistical computing. R Foundation for 225 Statistical Computing, Vienna, Austria. Available from https://www.R-project.org/

[CIT0013] Reuter R., Moffet C., Horn G., Zimmerman S., and Billars M.. 2017 Daily variation in intake of a salt-limited supplement by grazing steers. Prof. Anim. Sci. 33(3):372–377. doi:10.15232/pas.2016-01577.

[CIT0014] Wagnon K. A 1965 Social dominance in range cows and its effect on supplemental feeding, Berkeley (CA): Agricultural Experiment Station; p. ill (Bulletin819).

[CIT0015] White H. C., Van Emon M. L., Delcurto-Wyffels H. M., Wyffels S. A., and Delcurto T.. 2019 Impacts of form of salt-limited supplement on supplement intake behavior and performance with yearling heifers grazing dryland pastures. Transl. Anim. Sci. 3(Suppl. 1):1650–1654. doi:10.1093/tas/txz048. 32704930 PMC6999143

[CIT0016] Williams G., Beck M., Thompson L., Horn G., and Reuter R.. 2018a Variability in supplement intake affects performance of beef steers grazing dormant tallgrass prairie. Prof. Anim. Sci. 34(4):364–371. doi:10.15232/pas.2017-01720.

[CIT0017] Williams A. R., Wyffels S. A., Parsons C. T., Dafoe J. M., Boss D. L., Bowman J. G., Davis N. G., and DelCurto T.. 2018b The influence of beef cow weaning weight ratio and cow size on winter grazing and supplement intake behavior. Transl. Anim. Sci. 2(Suppl. 1):S84–S88. doi:10.1093/tas/txy045. 32704742 PMC7200815

[CIT0018] Wyffels S. A., Williams A. R., Parsons C. T., Dafoe J. M., Boss D. L., DelCurto T., Davis N. G., and Bowman J. G 2018 The influence of age and environmental conditions on supplement intake and behavior of winter grazing beef cattle on mixed-grass rangelands. Transl. Anim. Sci. 2:S89–S92. doi:10.1093/jas/skaa217. 32704743 PMC7200900

[CIT0019] Wyffels S. A., Parsons C. T., Dafoe J. M., Boss D. L., McClain T. P., Carter B. H., DelCurto T.. 2020 The influence of age and winter environment on Rumax Bovibox and Bovibox HM supplement intake behavior of winter grazing beef cattle on mixed-grass rangelands. Transl. Anim. Sci. (In press). 10.1093/tas/txaa093 PMC7754213 33381718

